# Root Tropisms: Investigations on Earth and in Space to Unravel Plant Growth Direction

**DOI:** 10.3389/fpls.2019.01807

**Published:** 2020-02-21

**Authors:** Lucius Wilhelminus Franciscus Muthert, Luigi Gennaro Izzo, Martijn van Zanten, Giovanna Aronne

**Affiliations:** ^1^ Department of Agricultural Sciences, University of Naples Federico II, Naples, Italy; ^2^ Molecular Plant Physiology, Institute of Environmental Biology, Utrecht University, Utrecht, Netherlands

**Keywords:** *Arabidopsis*, Cholodny-Went, directional growth, gravitropism, microgravity

## Abstract

Root tropisms are important responses of plants, allowing them to adapt their growth direction. Research on plant tropisms is indispensable for future space programs that envisage plant-based life support systems for long-term missions and planet colonization. Root tropisms encompass responses toward or away from different environmental stimuli, with an underexplored level of mechanistic divergence. Research into signaling events that coordinate tropistic responses is complicated by the consistent coincidence of various environmental stimuli, often interacting *via* shared signaling mechanisms. On Earth the major determinant of root growth direction is the gravitational vector, acting through gravitropism and overruling most other tropistic responses to environmental stimuli. Critical advancements in the understanding of root tropisms have been achieved nullifying the gravitropic dominance with experiments performed in the microgravity environment. In this review, we summarize current knowledge on root tropisms to different environmental stimuli. We highlight that the term tropism must be used with care, because it can be easily confused with a change in root growth direction due to asymmetrical damage to the root, as can occur in apparent chemotropism, electrotropism, and magnetotropism. Clearly, the use of *Arabidopsis thaliana* as a model for tropism research contributed much to our understanding of the underlying regulatory processes and signaling events. However, pronounced differences in tropisms exist among species, and we argue that these should be further investigated to get a more comprehensive view of the signaling pathways and sensors. Finally, we point out that the Cholodny-Went theory of asymmetric auxin distribution remains to be the central and unifying tropistic mechanism after 100 years. Nevertheless, it becomes increasingly clear that the theory is not applicable to all root tropistic responses, and we propose further research to unravel commonalities and differences in the molecular and physiological processes orchestrating root tropisms.

## Introduction

Although plants are sessile organisms, their organs including roots are not motionless. Movements of plant have fascinated scientists for ages (Whippo and Hangarter, 2006). This includes Charles Darwin who laid the foundations for accurate studies on movements of different plant organs in response to external directional stimuli, especially light and gravity (Darwin and Darwin, 1880). One particular type of plant movement are tropistic responses, defined as “a directional growth response to a directional stimulus” (Gilroy, 2008). Tropistic responses are distinguished from nastic responses by being directional relative to the stimulus. Tropisms can be classified as “positive” or “negative” according to the exhibited growth toward or away from the directional stimulus, respectively (Schrank, 1950; Gilroy, 2008).

**Figure 1:**
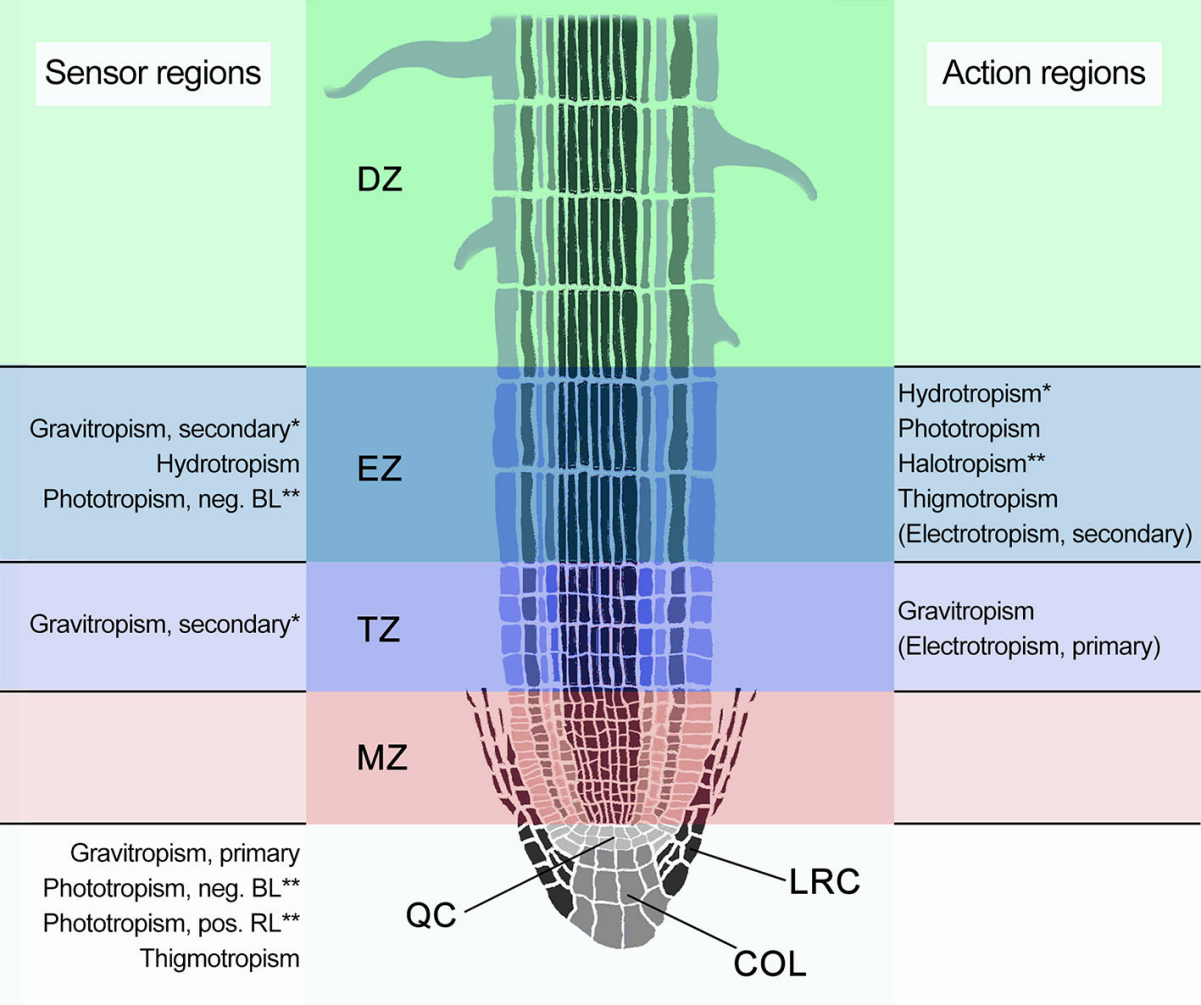
Schematic representation of a longitudinal cross section of an *Arabidopsis* root apex, indicating the four distinct developmental zones: the meristematic zone (MZ; pink), the transition zone (TZ; purple), also known as distal elongation zone (DEZ), the elongation zone (EZ; blue), and the differentiation zone (DZ; green). The root cap is indicated in gray and consists of the columella root cap (COL) and the lateral root cap (LRC) that, together with the MZ, surround the quiescent center (QC). Known or suspected sensor and action regions are indicated alongside the root. Tropisms within parentheses are likely not *sensu stricto* tropisms. BL, blue light; RL, red light. *Specific localization in the cortex of the EZ. **Suspected localizations.

Root tropisms are exerted through differentially-regulated cell growth on opposite sides of the root tip in specific root zones (Gilroy and Masson, 2008). Until the early 2000s the traditional anatomical view identified three main zones in the root tip, directly distal from the root cap: the root apical meristematic zone (MZ), the elongation zone (EZ), and the differentiation zone (DZ), which was based on the premise that cell elongation initiates immediately after the apical meristem (Dolan and Davies, 2004). However, evidence of a distinct cell population in the part of the EZ more distal from the base of the root has been presented in the last three decades. This region was dubbed distal elongation zone (DEZ) initially, and later transition zone (TZ), due to its unique characteristics (Ishikawa and Evans, 1993; Verbelen et al., 2006; Baluška et al., 2010). In the current view, four zones are thus identified, each characterized by specific cell types, cellular activities, and specific responses to tropistic signals (**Figure 1**, **Table 1**). The root cap consists of the columella and the lateral root cap surrounding the MZ, a zone of ​​active cell divisions which is followed by the TZ (**Figure 1**). The cells in the TZ undergo isodiametric cell growth with nuclei located in the center of the cells, similar to the meristem. Following the TZ, cells in the EZ rapidly elongate and nuclei are pushed toward the lateral cell walls due to the formation of large central vacuoles. Cells progressively slow down their elongation and finally reach their mature lengths within the differentiation zone (DZ), which is characterized by root hair development (**Figure 1**) (Verbelen et al., 2006).

**Table 1:** Root tropism sensor regions, signaling mechanism, and action regions in *Arabidopsis thaliana*.

Tropism		Sensor region	Signalling	Action region
*Gravitropism*	*primary*	Columella S1 and S2^[1]^	Cholodny-Went^[2]^	Basal TZ^[3]^
	*secondary*	TZ or EZ^[4]^	Likely not C-W^[5]^	Apical TZ^[4]^
*Hydrotropism*		EZ^[6]^	Likely not C-W^[7]^	EZ cortex^[6, 3]^
*Phototropism*	*BL neg.*	Likely EZ or root cap^[8, 9, 10]^	Likely not C-W^[11]^	EZ^[10]^
	*RL pos.*	Possibly root cap^[12, 13]^	Unknown	EZ^[12]^
	*BL pos.*	Unknown	Unknown	Unknown
*Halotropism*		Unknown	Cholodny-Went^[14]^	Likely EZ*^ [15, 16]^
*Thigmotropism*		Strongest in root cap^[17]^	C-W ^[18]^	EZ ^[17]^
*(Electrotropism^†^ )*	*primary*	Unknown	Unknown	TZ^[19]^
	*secondary*	Unknown	Unknown	EZ^[19]^
*(Thermotropism^†^ )*		Unknown	Unknown	Unknown
*Oxytropism*		Unknown	Possibly C-W^[20]^	Unknown
*(Phonotropism^†^ )*		Unknown	I.a. Ca^2+ [21]^	Unknown

*Despite a lack of direct reporting, the action region of halotropism is likely in the EZ, as it is a Cholodny-Went tropism.

^†^Likely not a sensu stricto tropism.

^[1]^(Blancaflor et al., 1998), ^[2]^(Geisler et al., 2014), ^[3]^(Krieger et al., 2016), ^[4]^(Wolverton et al., 2002), ^[5]^(Wolverton et al., 2011), ^[6]^(Dietrich et al., 2017), ^[7]^(Shkolnik et al., 2016), ^[8]^(Sakamoto and Briggs, 2002), ^[9]^(Briggs and Christie, 2002), ^[10]^(Mullen et al., 2002), ^[11]^(Kimura et al., 2018), ^[12]^(Kiss et al., 2003b), ^[13]^(Salisbury et al., 2007), ^[14]^(Galvan-Ampudia et al., 2013), ^[15]^(van den Berg et al., 2016), ^[16]^(Yokawa et al., 2014), ^[17]^(Massa and Gilroy, 2003), ^[18]^(Lee et al., 2020), ^[19]^(Wolverton et al., 2000), ^[20]^(Eysholdt-Derzsó and Sauter, 2017), ^[21]^(Rodrigo-Moreno et al., 2017).

In the late nineteenth and early twentieth century, several phenomenological studies on tropisms were conducted. During the final decades of the twentieth century, the focus moved to studies on the molecular mechanisms of root tropisms, enabled by new techniques in molecular genetics and supported in the first decade of the twenty-first century by special research environments such as the International Space Station (ISS) (Wolverton and Kiss, 2009; Kiss, 2015). Currently, research on plant tropisms becomes critical for advancing plant-based life support systems in space considering their fundamental role in producing fresh food and recycling of air and water (Lasseur et al., 2010). More in-depth knowledge of root growth response to a directional stimulus is required to design plant-based life support facilities able to guide root growth in a desired direction, as the gravity vector is absent in space. At the same time, the possibility of performing explorative experiments in the space environment, together with the development of new technologies, is also crucial to pave the way toward the goal of deepening our fundamental understanding of plant tropisms and their underlying molecular networks on Earth (Borst and van Loon, 2009; Gómez and Izzo, 2018).

Many different types of tropisms have been proposed over the years. Of these, gravitropism, phototropism, hydrotropism, halotropism, and thigmotropism are the most extensively studied. Physiological studies from around the turn of the twentieth century also investigated directional growth responses to electrical, chemical, and temperature gradients, among others (Bennett, 1904; Fitting, 1905). Some of these have received renewed attention in the 1990's, the most important of which being chemotropism, magnetotropism, electrotropism, and oxytropism. Whether these can be categorized as *bona fide* tropisms *sensu stricto* (i.e., directional growth responses to a directional stimulus (Gilroy, 2008) is in many cases still a matter of debate. However, it is certainly possible that more tropisms are still to be identified, as the recently proposed phonotropism illustrates (Rodrigo-Moreno et al., 2017).

In this review, an overview of all known and proposed tropistic responses with a focus on the roots is provided, and current insight into the different types of tropisms and their underlying molecular signaling mechanisms is discussed.

## Gravitropism

Our fundamental understanding of the reliable downward movement of plant roots is based on the Cholodny-Went theory (Cholodny, 1927; Went, 1928; Orbovik and Poff, 1993). Their central premise that a differential localization of auxin causes differential elongation still stands firm (Sato et al., 2015). According to this theory, accumulation of auxin in the root tip on the side closest to the direction of the gravity vector triggers a decrease in cell elongation within the basal zone of the root cap, causing the root to bend in the direction of the gravity vector (Geisler et al., 2014; Krieger et al., 2016).

An important elaboration on the Cholodny-Went theory is the auxin fountain model, that proposed how differential auxin levels in the root are established and regulated (Kramer and Bennett, 2006; Grieneisen et al., 2007; Mironova et al., 2012; Geisler et al., 2014). Most of the auxin in plant roots is synthesized in and around the columella cells (Petersson et al., 2009). According to the fountain model, auxin flows upward from these synthesis sites through the epidermis and partially flows back through the cortex, endodermis, and pericycle to the vasculature, where it returns to the root tip. When the root is not positioned in the direction of gravity, the auxin flow toward the basal oriented part is increased, while the flow to the adaxial parts decreases (Geisler et al., 2014; Swarup and Bennett, 2018). After gravitropic bending, not all plant roots are fully oriented in the direction of the gravity vector, but at various angles, based on the developmental stage and environmental circumstances. This fixed growth angle has been called the gravitropic set-point angle (GSA), which is at 0° when the root grows straight downwards (Digby and Firn, 1995).

Like in most responses to environmental signals, three distinct phases are typically recognized in the process of gravitropism: perception of the stimulus, signal transmission, and growth response (Toyota and Gilroy, 2013). Sensing of the gravity vector occurs in the columella cells, located in the center of the root cap (**Figure 1**). There, starch-rich amyloplasts, called statoliths, sediment in aggregates within the cell in response to gravity, due to their high mass (Leitz et al., 2009). The statoliths are free to sediment through the cytoplasm, in part because the nuclei are located at the top of the cells, the vacuoles are small, and because the endoplasmic reticula (ER) lie close to the plasma membrane (Morita and Tasaka, 2004).

As plastids, the amyloplasts possess a Translocon at the Outer Envelope Membrane of Chloroplasts (TOC) complex, which functions in gravitropism as well (Stanga et al., 2009). Disruption of the central pore protein, TOC75, or one of the receptor proteins, TOC132, strongly enhances the gravitropic deficiency of the *altered response to gravity (arg1)* mutant. ARG1 and its paralog ARG1-LIKE2 (ARL2) are type-II DnaJ-like peripheral membrane proteins and localize to the plasma membrane and the BFA sensitive endomembrane trafficking pathway (Boonsirichai et al., 2003; Harrison and Masson, 2008). While ARG1 is expressed throughout the whole plant, ARL2 is specifically expressed in the columella cells. Outside of the *arg1* mutant background, disruption of TOC132 or TOC75 does not, or only slightly attenuate gravitropism, respectively (Stanga et al., 2009). These findings suggest a role in the early gravitropic signaling for ARG1 and the TOC complex.

In accordance with the starch-statolith hypothesis, starchless *Arabidopsis thaliana phosphoglucomutase* (*pgm*) mutants displayed strongly reduced gravitropism (Caspar and Pickard, 1989). However, some gravitropic responsiveness remained in the *pgm* mutants, suggesting that statolith movement alone may not be sufficient to account for all gravity sensing (Caspar and Pickard, 1989; Kiss et al., 1989).

There are several theories about how the directional sedimentation of the statoliths affects processes in the cell to alter auxin flows (Strohm et al., 2012; Su et al., 2017). According to Leitz et al. (2009), the sedimentation of statoliths on the cortical ER causes ~200 nm indents, resulting in local expansion of the membrane surface of 15–20%. Mechanosensitive ion channels, particularly those for Ca^2+^, could be activated by this membrane distortion (Hamill and Martinac, 2001). The ER, where the statoliths sediment, is also a major storage compartment for Ca^2+^ (Urbina et al., 2006). This could connect the sedimentation of the statoliths to the later bi-phasic Ca^2+^ pulse characteristic of gravitropic signaling (Plieth and Trewavas, 2002). A detailed discussion of Ca^2+^ kinetics in gravitropism is summarized in Tatsumi et al. (2014).

The protoplast-pressure model is a modification of the ER membrane distortion theory, stating that the pressure of protoplast on the plasma membrane causes mechanosensitive ion channels to open, instead of local pressure exerted by statoliths (Wayne and Staves, 1996; Yoder et al., 2001; Perbal and Driss-Ecole, 2003). Statoliths do however add extra ballast to protoplasts, enabling the application of more pressure on the plasma membrane. Accordingly, 5*g* acceleration was sufficient to fully restore gravitropism after starchless *pgm* mutants were exposed to hypergravity conditions during centrifugation (Fitzelle and Kiss, 2001).

The alternative ligand-receptor interaction model adds to both the local and protoplast-pressure membrane distortion theories in explaining more directly how secondary messengers are activated. The ligand-receptor interaction model proposes that the contact between a ligand on the membrane of the statoliths and a receptor on the outer membrane of the ER results in the activation of cortical ER ion channels after sedimentation (Strohm et al., 2012). A promising candidate for interaction with this putative ER receptor was the TOC132 receptor protein, extending into the cytosol from the TOC complex on the statolith membrane (Stanga et al., 2009). However, the cytosolic domain of TOC132 turned out not to be necessary for a full gravitropic response (Strohm et al., 2014). Despite indications that the ligand-receptor model holds true for the alga *Chara globularis*, to the best of our knowledge, no evidence for the ligand-receptor interaction model has been presented in flowering plants so far (Braun, 2002).

Recently, the membrane phospholipid phospholipase C2 (PLC2) was shown to influence polar distribution of PIN2 in the early gravitropic signaling cascade (Chen et al., 2019). Gravitropic defects of *Arabidopsis* roots with inhibited PLC activity were previously reported, indicating that PLCs are involved in gravitropism (Andreeva et al., 2010). However, this was possibly due to these seedlings also displaying severe morphological and growth defects. PLC2 is known to produce the common secondary messenger inositol 1,4,5-trisphosphate (InsP_3_) and 1,2-diacyglycerol (DAG) from the hydrolysis of phosphatidylinositol 4,5-bisphosphate (PtdInsP_2_) (DeWald et al., 2001). InsP_3_ is involved in the early stages of gravitropism, before the establishment of the auxin asymmetry (Perera et al., 2006). By generating transgenic lines expressing human type I InsP 5-ptase, which hydrolyses InsP_3_, levels of InsP_3_ were reduced by at least 90%. This caused a decrease in establishment of auxin asymmetry, resulting in a slower and 30% decreased gravitropic response compared to wild-type *Arabidopsis* (Perera et al., 2006). Furthermore, InsP_3_ has been shown to influence gene expression in reaction to a gravitropic stimulus (Salinas-Mondragon et al., 2010). Of the downregulated genes, a substantial number is related to plastids and mitochondria. Of the upregulated genes, several are transcription factors and protein kinases linked to Ca^2+^ regulation (Salinas-Mondragon et al., 2010). This link between InsP_3_ and Ca^2+^ is corroborated by the observed close association between the two secondary messengers in both timing and effect in relation to PIN regulation (Zhang J. et al., 2011). However, although an InsP_3_-gated Ca^2+^ release channel in the ER membrane has been identified in mammalian cells, no such direct link has yet been found in plants (Zhang S. et al., 2011).

Changes in pH are also involved in the early gravitropic signaling. While the root cap apoplast pH decreased from 5.5 to 4.5, the pH of columella cell cytoplasm increased from 7.2 to 7.6 after gravitropic stimulation (Fasano et al., 2001). Preventing the pH increase of columella cytoplasm through the release of caged protons also delayed the onset of gravitropism. Mutants lacking *ALTERED RESPONSE TO GRAVITY (ARG1)* did not display this pH change in the root cap and show reduced and delayed gravitropism (Boonsirichai et al., 2003). Both ARG1 and plasma membrane H^+^-ATPases are localized to the plasma membrane and the BFA sensitive endomembrane trafficking pathway, which could be connected to the effect of ARG1 on cytoplasm pH (Boonsirichai et al., 2003).

Using the microgravity (µ*g*) environment of the ISS, the involvement of the cytoskeleton in gravitropism has been established. In microgravity, lentil (*Lens culinaris*) amyloplasts were clustered in the proximal part of the columella cells, which was contrary to the random distribution of amyloplasts in the plants grown on a clinostat; i.e., a rotating device used to simulate a low gravity environment for plant growth (Perbal and Driss-Ecole, 1989). This result indicated involvement of actomyosin in the positioning of amyloplasts, which was later corroborated (Driss-Ecole et al., 2000). It also showed that the randomization of the gravity vector achieved by the clinostat does not elicit the same effects as the -virtual- absence of the gravity vector in µ*g* conditions (Sievers and Hejnowics, 1992; Hoson et al., 1997). In microgravity conditions, statoliths do not have sedimenting amyloplasts. Thus these cells also lack an asymmetrical distribution of auxin in the root (Ferl and Paul, 2016). Several papers also indicated that actin in the cytoskeleton has a significant role in gravity signaling, as pressure exerted by sedimentation of statoliths on actin polymers could conduct a physical pressure signal toward the plasma membrane or ER membrane, causing ion channels to open (Yoder et al., 2001; Perbal and Driss-Ecole, 2003). Additionally, the ARP3 subunit of the Actin-Related Protein 2/3 (ARP2/3) complex is involved in regulating amyloplast sedimentation kinetics, as *Arabidopsis distorted1 (dis1)* mutants lacking ARP3 display a delayed response to gravitropic stimulation (Zou et al., 2016). However, the exact role of the cytoskeleton deserves more attention, as pharmacological experiments gave contradictory results, showing both inhibition and promotion of gravitropism (Ma and Hasenstein, 2006; Blancaflor, 2013). A detailed overview of studies of early gravitropic signaling is summarized in Nakamura et al. (2019).

Once perceived by the statoliths, the gravitropic signal generates a differential auxin distribution in the root. This process is dependent on the auxin influx carrier AUX1, which is expressed in the root tip and elongation zone (EZ, also known as the central elongation zone) (Marchant, 1999). Interestingly, recent experiments indicated that auxin is not only involved in the regulation of the gravitropic response, but also indirectly in gravitropic perception. Through the TIR1/AFB auxin receptor signaling pathway, auxin regulates the *PHOSPHOGLUCOMUTASE (PGM), ADENOSINE DIPHOSPHATE GLUCOSE PYROPHOSPHORYLASE (ADG)* and *STARCH SYNTHASE 4* (*SS4)* starch synthesis genes that are responsible for the establishment of statoliths in the cell (Zhang et al., 2019).

The change in auxin flow direction in roots that are not orientated toward the gravity vector is mediated by relocation of the PIN-FORMED3 (PIN3) and PIN7 auxin efflux carrier proteins (Friml et al., 2002; Kleine-Vehn et al., 2010). When the root is positioned vertically, these proteins are present at all sides of the columella cells. During gravistimulation, vesicles from endosomal compartments containing these PIN proteins relocate to the then lowest part of the cell, thereby providing increased efflux of auxin at that side, and decreased efflux on the opposite (upward oriented) parts of the cell (Geldner et al., 2001; Friml, 2010). For the relocation of PIN3 in the gravity sensing columella cells, ARG1 and ARL2 are necessary (Harrison and Masson, 2008).The innermost columella cells of the second tier are thought to have the most influence on the redirection of auxin (Blancaflor et al., 1998).

Next to roles for PIN3 and PIN7, changes in auxin flux affect the localization and degradation of PIN2 proteins that mediate the basipetal auxin flow. High auxin levels cause PIN2 proteins to be retained longer in the plasma membrane (Paciorek et al., 2005; Abas et al., 2006). Auxin has also been shown to increase proteasomal degradation of PIN2 proteins, suggesting a complex homeostatic mechanism that controls the extension of the polar auxin distribution from the columella cells to the EZ (Abas et al., 2006). Alongside increased auxin, PLC2 is also needed for proper retainment of PIN2 in the plasma membrane (Chen et al., 2019). Additionally, *plc2* mutants have reduced auxin content and reduced responsiveness to exogenous auxin. After relocation and degradation of PIN proteins in the root cap, the differential auxin distribution is extended toward the EZ, due to the auxin fountain mechanism (Grieneisen et al., 2007). In the basal part of the transition zone (TZ, also known as the distal elongation zone or DEZ), most of the gravitropic bending takes place in response to the auxin asymmetry (**Figure 1**) (Krieger et al., 2016).

Based on experiments where gravitropism was induced while the root tip was maintained at a constant angle against the gravity vector, Wolverton et al. (2002) proposed that a second gravity sensor could be located in the apical part of the TZ that contributes ~20% to the total gravitropic curvature (**Figure 1**). This has been called the “dual motors and sensors” theory. During gravistimulation, the electrical properties of the TZ changed markedly, indicating that this alternative gravity sensor could involve electrical signals (Ishikawa and Evans, 1990a; Collings et al., 1992). The presence of a second sensor and motor could also explain why *pgm1* mutants retained one third of the rate of wild type gravitropism, without a need for an auxin gradient (Kiss et al., 1996; Wolverton et al., 2011).

Ion channel activity of plant cells and their selective retention of charges cause electric currents in their cellular environment, which are altered by increased asymmetric proton efflux during gravitropism (Ishikawa and Evans, 1990a; Baluška and Mancuso, 2013). The electrical current density and orientation differ among different regions of maize (*Zea mays*) roots, as Collings et al. (1992) have noted. The TZ exhibits an inward oriented current, which is contrary to the outward orientation in the meristem, EZ, and basal end of the elongation zone. A similar pattern has been recorded for cress (*Lepidium sativum*) (Weisenseel et al., 1992). However, while the role of electrical currents in gravitropism is comparable, differences between species are apparent, as blocking of Ca^2+^ channels in maize had no effect on gravitropism, while limiting Ca^2+^ availability abolished gravitropism in cress (Collings et al., 1992; Weisenseel et al., 1992).

Within minutes after gravistimulation, the electrical current symmetry is disturbed. An increased proton efflux then creates a strong outward current at the upper surface of the horizontal root, near the root tip. In maize, this phenomenon has been observed at 1 to 2.5 mm from the root tip and in cress at 0 to 4 mm from the root tip (Collings et al., 1992; Weisenseel et al., 1992). This location partly corresponds to the location of the TZ. No basipetally propagating wave of proton efflux was detected in maize. Instead, it seemed that the efflux was synchronized in the youngest cells of the EZ, which is around 2.5 mm distal from the root tip in maize (Collings et al., 1992). While this increase in proton efflux in the TZ is linked by both Collings et al. (1992) and Weisenseel et al. (1992) to cell growth by apoplast acidification, the cytoskeletal rearrangements that are required for elongation are almost completely absent in the TZ. Because of this, the zone is also named the transition zone (TZ) instead of the distal elongation zone (Baluška et al., 2010; Baluška and Mancuso, 2013). As there is little elongation in the TZ, the bending in this second gravitropic motor and sensor region likely requires a different mechanism, as proposed by Wolverton et al. (2002). According to Baluška et al. (2010), the progression of cells into and through the TZ is decreased on the lower side and increased on the upper side of a horizontally oriented root. These differences in developmental speed then cause the root to bend at the TZ. Interestingly, the TZ has a peak of so called “brefeldin A (BFA)-induced compartments” that form because the BFA compound blocks endoplasmic reticulum to Golgi apparatus transport upon pharmacological application (Klausner et al., 1992). This peak precisely coincides with the location of TZ bending (Baluška et al., 2010). Since proper PIN2 localization also functions through a BFA-sensitive pathway, PIN2 retention in the TZ could have a critical role in the TZ gravitropic bending response (Abas et al., 2006).

In response to the established auxin asymmetry, root growth is altered asymmetrically. It is proposed that a large part of this change is caused by Ca^2+^ waves that elicit a change in pH (Monshausen et al., 2011). Within 2 to 6 min after reorientation, the upper flank epidermis experienced a Ca^2+^ level reduction and a pH decrease. The lower flank epidermis experiences the reverse, within the same time window (Monshausen and Sievers, 2002; Monshausen et al., 2011). The Ca^2+^ level reduction and a pH changes are likely connected to cell wall loosening, allowing for expansion when auxin levels are high (Monshausen et al., 2011). Gravitropic curvature in roots is also partially dependent on a transcriptional response to high auxin, enabled through decreased repression of auxin response factors (ARFs) by AUX/IAA proteins (Su et al., 2017).

Reactive oxygen species (ROS) have been shown to accumulate in root tips of gravistimulated maize in response to auxin (Joo et al., 2001). This accumulation was strongly reduced when phosphatidylinositol 3-kinase (PtdIns 3-kinase) activity was blocked, leading to reduced gravitropic reaction of the roots (Joo et al., 2005). A later report specified that the accumulation of ROS was higher at the concave or lower side of the root in the TZ after gravistimulation (Krieger et al., 2016). Other than the involvement of PtdIns 3-kinase and its product, phosphatidylinositol 3-phosphate (PtdIns3P), little is known about the asymmetric ROS gradient generated in response to gravitropically increased auxin levels.

Asymmetric increase of nitric oxide (NO) levels, centered around the TZ of the lower root side, is crucial for root gravitropism (París et al., 2018). When NO was reduced by adding a NO scavenger to the medium, cells of gravistimulated roots did not exhibit a PIN2 asymmetry in their plasma membranes. However, growth of *Arabidopsis* in general was also severely inhibited, prohibiting the drawing of sound conclusions on the specificity of NO effects on root gravitropism (París et al., 2018).

Similarly, an asymmetric increase of gibberellic acid (GA) levels is found at the lower side of gravistimulated roots (Löfke et al., 2013). Higher auxin levels at the lower side of the root cause a decrease in cycling of PIN2 to the lytic vacuole in the EZ (Kleine-Vehn et al., 2008). High GA levels seem to influence PIN2 retainment in the plasma membrane in the same way, by preventing PIN protein trafficking to the lytic vacuole (Löfke et al., 2013). The relative contribution to gravitropic bending in the EZ of this GA-mediated PIN2 stabilization, compared to the effects of auxin, has however not been determined yet.

Although gravitropism is the most studied tropism in plants, there are still important gaps in the knowledge of the signaling cascade. The sensory mechanism for primary gravitropism is known, but it remains largely elusive how the signal is transduced to InsP_3_ and later Ca^2+^ signals. Clearly, auxin asymmetry explains large parts of the gravitropic bending in the EZ. The picture is however complicated by the initiation of gravitropic curvature in the TZ. Additionally, the (possible) roles of various other signals, such as Ca^2+^, pH, ROS, NO, and GA levels, which are all to a certain extend altered asymmetrically in the EZ of gravistimulated roots, are poorly understood. Whether and how these signaling pathways connect to auxin signaling, or regulate gravitropic responses *via* parallel pathways, remains to be elucidated. Finally, as these secondary messengers are not necessarily confined to the columella, they could also constitute hubs for interaction of related tropism signaling pathways (**Table 2**) (Fasano et al., 2002; Salinas-Mondragon et al., 2010).

**Table 2:** Secondary messengers and phytohormones (potentially) involved in *Arabidopsis thaliana* root tropisms. With the following abbreviations: inositol 1,4,5-trisphosphate (InsP_3_), phospholipase Dζ2 (PLDζ2), phosphatidylinositol 3-phosphate (PtdIns3P), and phosphatidic acid (PA).

Tropism		Secondary messengers	Phytohormones
*Gravitropism*	*primary*	Ca^2+^ ^[1]^ , InsP_3_^[2]^, NO^[3]^, pH^[1]^ , PLDζ2^[4]^, PtdIns3P^[5]^, ROS^[6]^	Auxin^[7]^, gibberellic acid^[8]^
	*secondary*	Unknown	Unknown
*Hydrotropism*		Ca^2+^ ^[9]^, PLDζ2^[4]^, ROS^[6]^	ABA^[10]^, auxin?^[11,^ ^12]^, brassinosteroids^[13]^, cytokinin^[14]^, ethylene?^[15]^
*Phototropism*	*BL neg.*	Ca^2+^?^[16]^, Flavonoids^[17]^	Cytokinin^[18]^
	*RL pos.*	Unknown	Unknown
	*BL pos.*	Unknown	Unknown
*Halotropism*		Ca^2+^?^[19]^, Flavonoids^[20]^, H_2_O_2_?^[19]^, PLDζ2^[21]^	Unknown
*Thigmotropism*		Ca^2+^ ^[22]^, pH^[22]^, ROS^[22,^ ^23]^	Auxin^[24]^, ethylene^[25]^
*(Electrotropism^†^ )*		Unknown	Unknown
*(Thermotropism^†^ )*		Unknown	Unknown
*Oxytropism*		Unknown	Auxin?^[26]^, ethylene^[26]^
*(Phonotropism^†^ )*		Ca^2+^ ^[27]^, K^+^^[27]^, superoxide (O_2_^-^)^[27]^	Unknown

^†^Likely not a sensu stricto tropism.

^[1]^(Monshausen et al., 2011), ^[2]^(Perera et al., 2006), ^[3]^(París et al., 2018), ^[4]^(Taniguchi et al., 2010), ^[5]^(Joo et al., 2005), ^[6]^(Krieger et al., 2016), ^[7]^(Sato et al., 2015), ^[8]^(Löfke et al., 2013), ^[9]^(Takano et al., 1997), ^[10]^(Dietrich et al., 2017), ^[11]^(Shkolnik et al., 2016), ^[12]^(Kaneyasu et al., 2007), ^[13]^(Miao et al., 2018), ^[14]^(Saucedo et al., 2012), ^[15]^(Rowe et al., 2016), ^[16]^(Pedmale et al., 2010), ^[17]^(Silva-Navas et al., 2016), ^[18]^(Silva-Navas et al., 2016), ^[19]^(Shabala et al., 2015), ^[20]^(Petrella et al., 2018), ^[21]^(Galvan-Ampudia et al., 2013), ^[22]^(Monshausen et al., 2009), ^[23]^(Kurusu et al., 2013), ^[24]^(Lee et al., 2020), ^[25]^(Yamamoto et al., 2008), ^[26]^(Eysholdt-Derzsó and Sauter, 2017), ^[27]^(Rodrigo-Moreno et al., 2017).

The compounds thought to be involved on the basis of little or only indirect evidence, or with controversy, are followed by a question mark.

## Hydrotropism

Water acquisition is an important function of plant roots (Miyazawa et al., 2011). Because water availability in the soil is often spatially and temporally patchy, roots of many species can exert directional root growth toward water; i.e., hydrotropism. Even though hydrotropism has been described as early as 1887 (Von Sachs, 1887), the underlying mechanisms have not yet been fully elucidated (Eapen et al., 2005; Shkolnik and Fromm, 2016). One of the main reasons for this, is that gravitropism is often dominant over hydrotropic responses, making it difficult to study hydrotropism in isolation (Takahashi, 1997). The few reports published on hydrotropism in a natural environment have not observed a directional growth toward water where it was expected (Loomis and Ewan, 1936; Cole and Mahall, 2006). As Takahashi et al. (2009) proposes, this can be due to the balance between the influences of gravity and water being different between species. Hydrotropism has however been observed under lab conditions.

Hydrotropism appears not to function according to the Cholodny-Went theory, as no apparent changes in auxin distribution were observed in roots exhibiting hydrotropism (Shkolnik and Fromm, 2016; Shkolnik et al., 2016). Accordingly, pharmacologic application of auxin influx and efflux inhibitors did not affect hydrotropism, while it drastically decreased the root gravitropic responses (Shkolnik et al., 2016). However, application of the auxin antagonists α-(phenylethyl-2-one)-indole-3-acetic (PEO-IAA), auxinole, and the auxin response inhibitor PCIB gave contradicting results (Kaneyasu et al., 2007; Shkolnik et al., 2016). Possibly, components of auxin signaling are thus necessary, although hydrotropism may not depend on the establishment of an auxin gradient for differential growth *per se*.

Also contrary to the Cholodny-Went theory is the likely localization of both a hydrotropic sensor and response area in the EZ of *Arabidopsis* roots (**Figure 1**) (Krieger et al., 2016). While *de novo* gene expression in columella cells is not necessary for hydrotropism, laser ablation of stories 1 and 2 of the columella cells did severely decrease the hydrotropic response (Miyazawa et al., 2008). In contrast, preventing *de novo* gene expression in TZ cells did suppress hydrotropic curvature (Miyazawa et al., 2008). In a later study, laser ablation of the root meristem and columella cells had however no effect on hydrotropism in *Arabidopsis* (Dietrich et al., 2017). Possibly, multiple sensory regions for water gradients are present in *Arabidopsis* with the EZ and TZ seeming prominently involved.

Several *Arabidopsis* mutations have been identified that cause attenuation of the hydrotropic response; no hydrotropic response 1 (nhr1), mizu-kussei 1 (miz1), mizu-kussei 2 (miz2), and altered hydrotropic response 1 (ahr1) (Eapen et al., 2003; Kobayashi et al., 2007; Miyazawa et al., 2009a; Saucedo et al., 2012). The miz1 and miz2 mutants may be specifically disturbed in hydrotropic functioning, as they exhibited a normal response to gravity, and a wild type-like root cap organization. The highly conserved MIZ1 protein is likely located at the cytosolic side of the ER of columella cells and lateral root cap, as well as the TZ, but its molecular function remains unknown (Yamazaki et al., 2012). As miz1 roots show increased levels of auxin, it is thought that MIZ1 has a role in reducing auxin levels (Cassab et al., 2013). This effect of MIZ1 indicates that auxin levels are regulated in hydrotropism, although not asymmetrically (Dietrich, 2018).

The *miz2* mutation was identified as a weak *GNOM* mutant allele, involved in facilitating membrane trafficking (Geldner et al., 2003; Miyazawa et al., 2009b). No change in PIN1 localization was observed in *miz2* mutants, even though the ADP ribosylation factor guanine-nucleotide exchange factor (ARF-GEF) GNOM functions in the continuous recycling of PIN1 (Geldner et al., 2003; Miyazawa et al., 2009b). It has, therefore, been proposed that the effect of GNOM on hydrotropism may be distinct from its role in auxin distribution (Moriwaki et al., 2014).

In contrast to *miz1 and miz2*, little is known about the *ahr1* mutant, which displays no hydrotropism when confronted with a water gradient. The root meristem and EZ length, cell cycle duration, and primary growth of *arh1* mutants are not decreased following hydrotropic stimulation, as is the case for the wild type (Salazar-Blas et al., 2017). Upon addition of cytokinins, normal hydrotropic growth was restored in *arh1* mutants, indicating a critical role for cytokinins in hydrotropism (Saucedo et al., 2012).

Recently, Dietrich et al. (2017) identified a critical role for two subclass III Snf1-related kinases (SnRK2s) in the response of *Arabidopsis* to hydrotropic stimuli. SnRK2s function upstream of transcription factors in abscisic acid (ABA) phytohormone signaling (Cutler et al., 2010). While high ABA levels decrease root elongation, at low water potential, low ABA levels increase elongation (Rowe et al., 2016). Specifically, SnRK2.2 and SnRK2.3 play critical roles, as the *snrk2.2 snrk2.3* double mutant displayed severely inhibited hydrotropism. Strikingly, *SnRK2.2* and *MIZ1* expression is only needed in the cortex of the TZ and EZ (Dietrich et al., 2017). These results hint to a central role for ABA levels in the elongation and transition zone of the root cortex during hydrotropism, independent from the root meristem. A detailed overview of the components involved in hydrotropism is found in Cassab et al. (2013).

Using natural variation in hydrotropic responses among *Arabidopsis* accessions, Miao et al. (2018) identified H^+^ efflux near the root tip as an indicator for hydrotropism. Increases in H_2_O_2_ flux and Ca^2+^ influx in the same root region during hydrotropism were only observed in the strongly hydrotropic Wassilewskija (Ws) accession. Transcriptomic analysis indicated an important role for brassinosteroids and epigenetic regulation in hydrotropism in this accession. Indeed, the strong hydrotropic response of Ws was reduced when brassinosteroid perception was partially deficient. Increased activity of brassinosteroid-activated plasma membrane H^+^-ATPases was likely linked to the increased H+ efflux of Ws during hydrotropism. Although an increase of brassinosteroid levels was assumed from the expression of a brassinosteroid biosynthesis control gene, the actual brassinosteroid levels, as well as the function and localization of H^+^, Ca^2+^, and ROS during hydrotropism remains to be investigated.

The plasma membrane-associated cation-binding protein 1 (PCaP1) potentially functions as a signaling hub in hydrotropism (Tanaka-Takada et al., 2019). This protein is capable of binding Ca^2+^, Ca^2+^/calmodulin, and PtdInsP_2_ and is usually stably associated with the plasma membrane, despite that the protein lacks a transmembrane domain. During hydrotropism, PCaP1 localization in the EZ shifted to the cytoplasm. While hydrotropic bending is controlled in the EZ cortex, the change in PCaP1 localization is especially apparent in the endodermis (Dietrich et al., 2017). This position, combined with the initial membrane localization and the ability to bind Ca2+, points toward a potential central role in the hydrotropic signal transduction pathway. While it is also able to bind the InsP3 precursor PtdInsP_2_, no role for InsP_3_ has been confirmed in hydrotropism. It is also possible that the InsP_3_ binding capability represents a link to the gravitropic signaling mechanism, which needs to be suppressed before hydrotropic bending can take place.

Our understanding of hydrotropism is not as advanced as that of gravitropism, while significant interaction between the tropisms are apparent (Takahashi, 2003). One of the remaining open questions is how asymmetric signals are formed in the root in response to water patchiness and how these signals are transduced. The natural variation in hydrotropic competence of *Arabidopsis* accessions provide a valuable resource for hydrotropism research, in addition to the four known hydrotropic mutants (Miao et al., 2018). Additionally, experiments in space allow for the investigation of hydrotropic signaling without the interference from gravitropism.

## Phototropism

Plants evolved the ability to sense—and respond to—different characteristics of light, such as quantity, quality, duration (photoperiod), and direction, which is mediated by specialized photoreceptor proteins (Galvão and Fankhauser, 2015). Shoots and/or leaves of many plant species can optimize the amount of energy perceived through directional growth when exposed to non-uniform light conditions; called phototropism (Liscum et al., 2014). Already in the nineteenth century it was recognized that roots of some species grow away from light, while others grow toward the light (Von Sachs, 1868). The first response is known as negative phototropism, the second as positive phototropism.

Light conditions perceived in the shoot can also influence root growth and development *via* e.g., the master photomorphogenesis repressor COP1, influencing root apical meristem proliferation through modulation of PIN1 and PIN2 distribution (Sassi et al., 2012; van Gelderen et al., 2018). Roots can be exposed to light directly as well, despite their underground localization. Not only can light penetrate up to a few centimeters in the upper layers of some soils (Mandoli et al., 1990), the plant itself can also guide light through the stem to the roots due to the “stem pipe effect” (Mandoli et al., 1984; Lee et al., 2016). Aside from the above-mentioned phenomena, roots can also be exposed to light shortly after germination in the top layer of the soil or because cracks in the soil emerge that trigger a phototropic reaction. The precise evolutionary function of phototropism in roots is still under debate, although an increased root efficiency and enhanced seedling survival under dry conditions have been suggested as fitness benefits to the plant (Galen et al., 2007; Kutschera and Briggs, 2012).

Some of the principles and signaling pathways involved in the well-studied shoot phototropism responses also account for root responses to light (Esmon et al., 2005; Briggs, 2014). However, there are also clear differences, as for instance shoots, but not the roots, display distinctly different phototropic reactions to low fluence rate and high fluence rate light exposure (Parks et al., 2001). Moreover, the blue light photoreceptor PHOTOTROPIN2 (PHOT2/NPL1), important for high fluence light shoot phototropism, does not appear to be present in the root (Sakai et al., 2001; Kong et al., 2006). In addition, over 3,000 light-responsive genes are differentially expressed between hypocotyls and roots of *Arabidopsis* seedlings (Ma et al., 2005). A recent discussion of root and shoot phototropism in response to blue light is provided in Morrow et al. (2018).

Roots of many species respond with positive or negative phototropic growth to red and blue light, while others are insensitive. Early studies demonstrated that roots of about half of the tested species (circa 292) did not react to unidirectional white light, while the other half showed negative phototropism, and only a handful of species displayed a positive response (Hubert and Funke, 1937; Kutschera and Briggs, 2012). Most recent work focused on *Arabidopsis,* which mainly displays a negative blue light root phototropism (Zhang et al., 2013). The difference between plant species could be caused by the absence or presence of a functional phototropic mechanism for a specific part of the light spectrum, by a different light intensity threshold, or by a difference in balance between responses to diverse tropistic stimuli. Roots of individuals of the same species likely react similarly to light stimuli. Still, Kutschera and Briggs (2012) noticed distinct groups of cress reacting with positive, negative or no phototropism. However, these seedlings were grown in hydroculture, which constitutes a potentially detrimental flooding-like condition (Ashraf, 2012; Sauter, 2013). Indeed, Hubert and Funke (1937) had already rearranged their experimental setup after noticing such damaging effects of hydroculture on roots and found no differences in phototropic response of different cress individuals.

Some researchers have advocated for interpreting the far more abundant negative tropistic reaction to light as a stress reaction (Yokawa et al., 2014). Negative phototropism combined with increased root growth would then constitute an “escape tropism” (Yokawa et al., 2013). As an increase in ROS is part of several stress responses, the increase of ROS in illuminated roots is seen as an indication that root illumination can be considered a stress condition, justifying the term “escape tropism.” However, ROS is also an important part of gravitropic signaling (Krieger et al., 2016). Therefore, the ROS increase under light could represent regular physiological phototropic signaling rather than a stress indicator.

Different light sensors and signaling pathways are in place that mediate blue light and red light phototropisms (BLPT, RLPT) (Goyal et al., 2013). PHOTOTROPIN1 (PHOT1/NPH1) is a sensor for BLPT in roots and is, in *Arabidopsis*, predominantly localized in the internal tissues of the EZ (**Figure 1**) (Liscum and Briggs, 1995; Briggs and Christie, 2002). Upon blue light stimulation of *Arabidopsis* roots, PHOT1 is autophosphorylated at the plasma membrane and around 20% dissociates from the membrane (Sakamoto and Briggs, 2002; Knieb et al., 2004). In maize, only local root cap illumination is able to achieve white light-induced phototropic curvature in the EZ (Mullen et al., 2002). Therefore, it is possible that the expression pattern of phot1 is different in maize, or there is an unknown link between the root cap and PHOT1 in the EZ.

Despite the clear role for phototropins, the BLPT signaling cascade has not been fully elucidated. Following autophosphorylation, PHOT1 binds to PHYTOCHROME KINASE SUBSTRATE 1 (PKS1) together with ROOT PHOTOTROPISM2 (RTP2), a membrane-bound putative scaffolding protein (Inada et al., 2004; Boccalandro et al., 2008). NON-PHOTOTROPIC HYPOCOTYL 3 (NPH3) is dephosphorylated by blue-light-activated PHOT1, which functions as a substrate adapter for a CULLIN3-based E3 ubiquitin ligase (CRL3) (Pedmale and Liscum, 2007; Roberts et al., 2011). Under low-intensity blue light, this CRL3-NPH3 complex mono- or multiubiquitinates PHOT1, which could be connected to PHOT1 dissociation from the plasma membrane (Knieb et al., 2004; Roberts et al., 2011). Under high-intensity blue light, PHOT1 is polyubiquitinated, marking it for 26S proteasome-mediated degradation. This likely functions as a mechanism of receptor desensitization (Roberts et al., 2011).

One prevalent model connected PHOT1 activation to asymmetrical PIN2 distribution through altered trafficking (Wan et al., 2012). In this model, NPH3 functions as a point of interaction for gravitropic and phototropic signaling, that influences PIN2 distribution. In addition, PIN3 polarization is influenced through a GNOM-dependent trafficking pathway (Zhang et al., 2013). By changing the polarity and symmetrical distributions of PIN2 and PIN3, BLPT could function according to the Cholodny-Went theory, through the generation of auxin asymmetry (Pedmale et al., 2010; Zhang et al., 2014). However, a recent study by Kimura et al. (2018) presented critical notes to this model. An asymmetrical increase in auxin was found on the illuminated side of the root, in agreement with some earlier studies (Zhang et al., 2013; Zhang et al., 2014). However, Kimura et al. (2018) attests that this is a gravitropic reaction following the initial phototropic bending. Due to the BLPT-driven reorientation of the root, gravitropism would be activated, generating auxin asymmetry and opposing phototropic curvature. Inhibition or attenuation of auxin production and transport using pharmacological and genetic experiments was also found to increase BLPT, as it obstructed gravitropism (Kimura et al., 2018). These results suggest that auxin asymmetry may not be necessary, but instead antagonistic for establishing phototropic curvature in the root.

One possible mechanism involved in phototropism is the increase of flavonols in the TZ of the illuminated side of the root (Silva-Navas et al., 2016). This establishment of an asymmetric gradient of flavonols (e.g., quercetin and kaempferol) affects auxin signaling, PLETHORA gradient, and superoxide radical content. The resulting reduction of cell proliferation in the illuminated side of the root then causes curvature. Furthermore, cytokinin could be involved through regulation of flavonol biosynthesis, as the cytokinin receptor *cre1 ahk1* double mutant displayed reduced BLPT and flavonol accumulation (Silva-Navas et al., 2016).

Recently, a previously unknown positive blue-light phototropic response was identified in *Arabidopsis* in a microgravity environment (Vandenbrink et al., 2016). The response was only detectable at gravity levels below 0.3*g* and already attenuated around 0.1*g*. In addition, pre-treatment with 1 h of red light enhanced the positive blue light phototropism (Kiss et al., 2012; Vandenbrink et al., 2016). As both the *phyA* and *phyB* mutants displayed wild type-like curvature, it is likely that another phytochrome is responsible for this red-light mediated enhancement (Vandenbrink et al., 2016). Candidates include phyD and phyE, being both highly expressed in the root tip, with phyD also being expressed throughout the EZ (Salisbury et al., 2007).

In addition to blue light, *Arabidopsis* roots also respond to unilateral red light with positive tropistic curvature. For this positive red light phototherapy (RLPT) it is also necessary to attenuate gravitropism, either through rotation on a so called “ROTATO” feedback system, that keeps the root tip aligned with the gravity vector based on rotation after image processing and feedback, the use of a mutant (e.g., *pgm1*) or microgravity conditions (Ruppel et al., 2001; Kiss et al., 2003a; Vandenbrink et al., 2016). Interestingly, positive RLPT has an inverse relationship with the strength of gravity, in contrast to the apparent 0.1–0.3*g* threshold for positive BLPT (Vandenbrink et al., 2016). Mutations in *phyA* and *phyB* only partially inhibited the RLPT response, indicating a possible additive effect of phyA and phyB in RLPT (Kiss et al., 2003b; Kiss et al., 2012; Vandenbrink et al., 2016). The location of positive red-light phototropic curvature was found to be at the basal edge of the EZ (**Figure 1**) (Kiss et al., 2003b). PKS1 is one of the few proteins known to be involved in the process (Molas and Kiss, 2008). Under red light exposure PKS1 expression is increased in a phyA-dependent manner (Boccalandro et al., 2008). However, experiments with *phyA/B pks1* double mutants indicate that the function of PKS1 in RLPT is separate from both phytochromes. In addition, overexpression of PKS1 led to negative curvature in response to unilateral red light (Molas and Kiss, 2008). Whether or not red-light phototropism functions according to the Cholodny-Went theory and how it interacts with blue light phototropism remains to be studied. Based on the latent periods, negative BLPT has been proposed as relatively stronger than positive RLPT, with gravitropism stronger than both (Kiss et al., 2003a; Kiss et al., 2003b). Positive BLPT was only detected in microgravity and would most likely be of similar strength to RLPT, based on the comparable latent periods (Vandenbrink et al., 2016).

The most pressing issue in the study of phototropism has become the basic signal asymmetry causing the growth asymmetry, due to the findings of Kimura et al. (2018), which were critical of the assumed auxin driven explanation of phototropism. Flavonols and cytokinins provide a possible alternative signal gradient in this regard (Silva-Navas et al., 2016). While attenuation of gravitropism has provided insight in positive BLPT and positive RLPT in *Arabidopsis*, other species with higher phototropic competence would likely more suitable for experimentation on these subtle tropisms (Hubert and Funke, 1937).

## Halotropism

High levels of salt are detrimental for growth in most plant species. Plants respond to high salinity by extrusion of salt ions, sequestration, changes in root system architecture, and halotropism (Maathuis et al., 2014; Julkowska and Testerink, 2015). When confronted with a NaCl gradient, *Arabidopsis* roots can change their growth direction (Sun et al., 2008). This does not seem to be due to osmotic effects, as roots did not bend in response to a non-ionic osmotic mannitol gradient as high as 400 mM (Galvan-Ampudia et al., 2013). In most species, halotropism is negative (i.e., away from the directional stimulus), however, also species with positive halotropism have been identified. The halophyte *Bassia indica* for instance, displayed increased horizontal root growth toward a higher salt concentration when confronted with a salt gradient (Shelef et al., 2010).

In order to display halotropism, gravitropic growth must be attenuated. For *Arabidopsis,* the halotropic threshold lies between 50 and 100 mM NaCl (Sun et al., 2008; Galvan-Ampudia et al., 2013). At higher concentrations, the suppression of gravitropism becomes dose-dependent, with 85% of wild-type seedlings showing agravitropic root growth at 150 mM NaCl (Sun et al., 2008). One proposed manner by which halotropism can override gravitropism is the degradation of amyloplasts in the columella (Sun et al., 2008). Without a gravity signal, PIN2 internalization and proteolysis could be suspended, allowing for halotropic signaling, which functions primarily through altered PIN2 trafficking as well (Abas et al., 2006). The salt stress induces increased clathrin-mediated endocytosis of PIN2 in the root tip (Galvan-Ampudia et al., 2013; Zwiewka et al., 2015). If the root is presented with a NaCl gradient, PIN2 endocytosis increases more at the side of the root exposed to the higher NaCl concentration, which depends on phospholipase Dζ2 (PLDζ2) (Galvan-Ampudia et al., 2013). Under NaCl stress, the asymmetrically increased internalization of PIN2 from the plasma membrane causes an asymmetric flow of auxin in the root, which causes halotropic bending.

The increased internalization leads to a decrease in PIN2 abundance at the plasma membrane under severe salt stress (150 mM NaCl) (Sun et al., 2008). At the same time, PIN2 transcript levels decrease in root cells, only to be restored 8 h later (Sun et al., 2008). Even though this restoration has no perceived influence on PIN2 abundance at the plasma membrane, it coincides with the onset of halotropic curvature. Modelling predicted that through PIN2 internalization an auxin level increase of only 12–14% can be obtained at the non-stressed side of the root. This is well below the 30–40% estimated from observations (van den Berg et al., 2016). Increased PIN2 endocytosis alone is therefore likely not sufficient to explain halotropic growth.

No NaCl sensor has been conclusively identified yet, and one possibility is that instead of a discrete sensor, biophysical alterations trigger halotropic growth. This could be in the form of changes in plasma membrane tension due to saline conditions, which are able to directly change the endocytic cycling of auxin transporters, among which PIN1 and PIN2 (Zwiewka et al., 2015). Alternatively, the SALT OVERLY SENSITIVE (SOS) pathway could play a significant role in sensing NaCl concentrations. The *sos1-1, sos2-1, and sos3-1* mutants showed stronger agravitropic growth than wild-type when grown in saline conditions, despite these lines exhibiting slower amyloplast degradation (Sun et al., 2008). Additionally, there was no *PIN2* transcript level decrease in *sos1-1* mutants as seen in wild-type *Arabidopsis* under salt stress (Sun et al., 2008). The SOS pathway is therefore proposed to be critical for the early stages of halotropism.

Although involvement of auxin transporters other than PIN2 in halotropism has been suggested, only the effects of AUX1 and PIN1 have been corroborated experimentally. The auxin asymmetry generated by salt-induced increases in PIN2 internalization, combined with an asymmetric AUX1 pattern and a transient increase of PIN1 protein levels, could be sufficient in accounting for the total perceived auxin asymmetry (Galvan-Ampudia et al., 2013; van den Berg et al., 2016). Because the halotropic changes in AUX1 localization occur in the EZ and not near the root tip, it is possible that PIN2 is sufficient to explain the establishment of auxin asymmetry in halotropism. While in line with the Cholodny-Went theory, this would distinguish halotropism from the gravitropic PIN3 and PIN7-dependent establishment of auxin asymmetry (van den Berg et al., 2016).

Han et al. (2017) suggested possible involvement of the ATP BINDING CASSETTE-B (ABCB) transporters, PROTEIN PHOSPHATASE 2A (PP2A), and flavonoids in an elaborated halotropism model. Of the ABCB transporters present in *Arabidopsis*, ABCB1, ABCB4, and ABCB19 are known to use ATP hydrolysis to perform active auxin transport and mutants are affected in tropistic reactions (Peer et al., 2011). PP2A activity is regulated by phosphatidic acid (PA), which is a product of PLD and central to PIN2 recycling (Gao et al., 2013). Therefore, it is possible that PP2A regulates halotropism by dephosphorylating ABCB's and/or PIN2 (Han et al., 2017). The potential involvement of flavonoids in halotropism is inferred from their capacity to prolongate auxin signals and possible inhibition of ABCB transporters (Peer and Murphy, 2007). In addition, flavonoid production increases under saline conditions (Yan et al., 2014). Recent investigations have also revealed an important role for light in modulating root halotropism. While for *Arabidopsis* halotropic growth in the dark is more pronounced, rough bluegrass (*Poa trivialis*) show no halotropism without blue light (Yokawa et al., 2014; Petrella et al., 2018).

Despite its recent characterization, substantial progress has been made regarding the functioning of halotropism. Two of the main challenge are the identification of the halotropic sensor and the role of the SOS pathway. There are also strong indications of a link between halotropism and light or phototropism. As seen in rough bluegrass, halotropism can be conditional on illumination. Flavonoids, of which production increases under salt stress, contain a subset of flavonols that form a gradient in phototropism. Salinity, especially when combined with high light intensity, is also connected to increased ROS production, which could be involved in the tropistic reactions to these stimuli (Miller et al., 2010). With the projected increase of droughts due to climate change, exploration and exploitation of the link between salt and light responses could prove valuable for improving drought tolerance of crop species.

## Thigmotropism

Plant roots respond distinctly to touch signals, after encountering an obstacle in the soil (Monshausen and Gilroy, 2009). When plant roots encounter an obstacle in their growth path, the root first continues growing in the same direction, until slippage occurs when stored extension growth is released sideways. After the initial undirected slipping, the root produces a first bend in the basal end of the EZ, followed by a second bend in the TZ (**Figure 1**) (Massa and Gilroy, 2003). This second bending occurs in the opposite direction to the first one, creating a step-like shape with the largest part of the EZ horizontal, but the root cap again vertically oriented. This allows the root to grow sideways, circumventing the obstacle, while at the same time the root cap stays in touch with the surface of the obstacle, providing continuous tactile information about the blockade (Massa and Gilroy, 2003).

When a root is touched once, it elicits a single Ca^2+^ spike, while bending elicits a characteristic biphasic Ca^2+^ response (Monshausen et al., 2009). While most, if not all, regions of the root are touch-sensitive, the root cap is considered the site where perceived mechanical signals lead to a thigmotropic reaction. Resting cytosolic Ca^2+^ levels in root cap cells are lower than in other root cells, while touch stimulation of the cap elicits a higher Ca^2+^ spike (Legué et al., 1997).

Recently, the thigmotropic response was shown to be dependent on asymmetrical auxin distribution (Lee et al., 2020). When touching an obstacle during vertical growth, the root bends and auxin accumulates at the concave or higher side of the root. This auxin asymmetry is likely mediated by PIN2 asymmetry near the root tip (Lee et al., 2020). As gravitropic auxin asymmetry would be the opposite of the one found for thigmotropism, it is necessary that gravitropism is attenuated. Possibly, this is achieved through the decrease of amyloplast sedimentation rates in columella cells. This decrease in sedimentation is stronger after touch stimulation of the root cap than after touch stimulation elsewhere in the root (Massa and Gilroy, 2003).

Although the root cap is considered the most likely location where thigmotropic signaling originates, determining the actual sensory mechanism presents a sizable challenge [for an overview of mechanoperception models, see: (Fasano et al., 2002; Telewski, 2006)]. Possible receptors are: MECHANOSENSITIVE CHANNEL OF SMALL CONDUCTANCE proteins (MscS), MID1-COMPLEMENTING ACTIVITY (MCA) proteins, Piezo proteins, and RECEPTOR-LIKE KINASES, that monitor cell wall tension (Kurusu et al., 2013; Monshausen and Haswell, 2013). MCA1, a stretch-activated Ca^2+^ membrane channel protein, is a promising candidates as roots of *mca1*-null mutants are unable to penetrate a harder medium if allowed to grow on a softer medium first (Nakagawa et al., 2007). However, *mca1*-null mutants grown in harder medium from the start have a growth pattern and penetration ability similar to the wildtype (Nakagawa et al., 2007).

The signaling cascade connecting thigmotropic sensing to asymmetric PIN2 distribution is largely unknown, although several secondary messengers or cellular response candidates besides Ca^2+^ have been proposed. Cell alkalization, reactive oxygen species (ROS), and ethylene are all involved in the signaling or modulation of thigmotropic reactions (Yamamoto et al., 2008; Monshausen et al., 2009; Ponce et al., 2017). The extracellular pH of epidermal cells of the EZ and DZ increased by up to three pH units when touched, with no clear refractory period. This pH change was accompanied by a simultaneous yet smaller cytosolic pH decrease (around 0.2). The pH change did not spread to adjacent cells (Monshausen et al., 2009). Upon touch, a short (1 to 2 min) burst in ROS production was also noted. Interestingly, this ROS production and the resulting thigmotropic bending is severely decreased in the hydrotropic *ahr1* mutant. These observations suggest that thigmotropism and hydrotropism (and possibly other tropistic signals) cross talk at the level of AHR1 (Ponce et al., 2017). Further characterization of the *ahr1* mutant can, therefore, be key to study the currently underexplored interactions between tropisms and their relative strengths.

Both the alkalization and the increase in ROS production are caused by the influx of Ca^2+^ into the cell, possibly enhanced by release of Ca^2+^ from intracellular stores (Monshausen et al., 2009). The slower amyloplast sedimentation rate in response to touch also indicates involvement of the columella cell cytoskeleton (Massa and Gilroy, 2003). Yet how these changes interact and cause the PIN2 asymmetry leading to thigmotropic bending or attenuate gravitropic signaling is not fully understood.

As mentioned, the gaseous phytohormone ethylene is important for thigmotropism (Yamamoto et al., 2008). Roots suddenly encountering a rigid medium produced less ethylene and were more likely to bend than controls grown in only soft medium. Indeed, content of the ethylene precursor 1-aminocyclopropane-1-carboxylic acid (ACC) was reduced in roots shortly after contact with the rigid medium. The resulting lower ethylene levels also softened the root tip, which could help the root slip or bend. In contrast, roots that did not bend displayed enhanced levels of ethylene and had harder root tips, presumably to allow for better medium penetration (Yamamoto et al., 2008). Ethylene could also be involved in counteracting the effects of gravitropic auxin redistribution, as it is known to decrease cell elongation in roots in darkness (Le et al., 2001).

With the thigmotropic response likely functioning according to the Cholodny-Went theory, the challenge now is tying together the molecular connections of the various signals involved in the asymmetrical distribution of PIN2 (Lee et al., 2020). Attenuation of the gravitropic influence on the auxin asymmetry in thigmotropism could function through decreased amyloplast sedimentation, although this hypothesis needs to be confirmed. It is thus possible that there are multiple ways of attenuating gravitropism in Cholodny-Went tropisms, with the proposed mechanism of amyloplast degradation in halotropism also targeting the gravitropism sensor (Sun et al., 2008).

## Chemotropism

Various publications list chemotropism as one of the possible tropisms affecting root growth (Eapen et al., 2005; Bisgrove, 2008; Baluška et al., 2009; Barlow, 2010; Henke et al., 2014; Kordyum, 2014). The ability to induce changes in root growth toward or away from a chemical indeed could be considered a useful adaptation. For instance, nutrient-imposed redirection of root growth toward nutrient-rich parts of the soil can help in maximizing nutrient acquisition, especially in poor soils, while growing away from toxic compounds can help in tolerating poisonous soils. Directional growth toward nutrients has been the focus of most chemotropism research and has become the operational definition in most literature. However, in the strict definition, halotropism can be considered a chemotropic reaction as well.

Despite the intuitive importance of positive root chemotropism, only a handful of studies have presented experimental evidence for the existence of chemotropic mechanisms. Most recent studies into the nutrient acquisition strategy of plants focused on the establishment and developmental plasticity of root architecture (Campbell et al., 1991; López-Bucio et al., 2003; Hodge, 2004; Niu et al., 2013). Filleur et al. (2005) however showed that primary *A. thaliana* root angle was different between media with uniform sufficient (2 M) potassium and uniform low (0.05 M) potassium conditions. While this indicates that potassium has an effect on the direction of the primary root, the uniform exposure precludes it being a “true chemotropic” effect, but rather classifies it as a chemonastic movement (Kellermeier et al., 2014).

In addition to the positive *vs*. negative distinction in tropisms, Filippenko (2001) argues for a further division in active and passive tropistic reactions. An active chemotropic reaction for instance would be the sensing of a nutrient by the plant, followed by directional growth toward the nutrient. Passive chemotropic reactions includes responses to harmful heavy metals, because heavy metal salts such as cadmium nitrate [Cd(NO_3_)_2_] are not necessarily sensed by the plant but instead cause direct physical damage to the root cells or even necrosis, which results in a growth differential between the exposed and non-exposed sides of the root (Hasenstein and Evans, 1988; Wilkinson et al., 1991; Filippenko, 2001).

Concrete evidence for the influence of a differentially distributed nutrient on the directional growth of a primary root was provided more than a century ago by Newcombe and Rhodes (1904). A positive bending response toward disodium phosphate (Na_2_HPO_4_) of the root tips of white lupin (*Lupinus albus*) was observed, at concentrations of 0.28%. Higher concentrations (1 or 1.5%) of disodium phosphate resulted in the same directional growth response, followed by root necrosis. Seemingly, not all species exhibit chemotropism in the same manner, as roots of *Cucurbita pepo* displayed no preferential bending when exposed to a directional disodium phosphate stimulus (Newcombe and Rhodes, 1904). Other experiments with potassium nitrate (KNO_3_), magnesium sulphate (MgSO_4_), and calcium nitrate [Ca(NO_3_)_2_] reported in the study failed to trigger a chemotropic reaction in both *L. albus* and *C. pepo*. However, this study was criticized by Gilroy and Masson (2008) for not constituting a “robust chemotropic directional assay” as there was no repositioning of the stimulus. The positive chemotropic effect of disodium phosphate is supported by a recent experiment, where it was elicited in carrot seedlings (*Daucus carota*) onboard the ISS (Izzo et al., 2019). When confronted with both a hydrotropic and chemotropic stimulus under microgravity conditions, the roots grew preferentially into the substrate containing disodium phosphate. On the ground, both stimuli were overruled by gravitropism (Izzo et al., 2019). To the best of our knowledge, there is no information available on the underlying mechanisms, or the possible involvement of signal molecules.

## Magnetotropism

Magnetic fields both weaker and stronger than the geomagnetic field have distinct influences on plants (reviewed in Maffei, 2014). Research into magnetotropism departed as an experimental tool for elucidating the gravitropic mechanism (Audus, 1960; Belova and Lednev, 2000; Galland and Pazur, 2005). It became evident however, that a magnetic field itself could change the growth direction of primary roots and it was immediately presumed to function through the then already proposed starch-statolith hypothesis of gravitropism (Audus, 1960). Due to the markedly different magnetic properties of the amyloplasts compared to the surrounding cytoplasm and tissues, magnetic fields are able to redirect amyloplast sedimentation independent of the gravity vector. Magnetic fields thus can overcome gravitropism and the root is guided in the direction of magnetic attraction of the amyloplasts. This mechanism of magnetotropism has been corroborated by later research (Kuznetsov and Hasenstein, 1996; Kordyum et al., 2005; Bilyavska and Polishchuk, 2014). Interestingly, Pittman (1962, 1970) has shown that roots of oat (*Avena fatua*) and bread wheat (*Triticum aestivum*) align in a magnetic north-south direction, both in the field and in laboratory conditions. The magnetotropism trait was even claimed to be inheritable through the cytoplasm in *T. aestivum* cultivar crosses (McKenzie and Pittman, 1980). However, studies on magnetotropism have been confined to the phenomenological level only. No alternative has been explored to the idea that magnetotropism is merely a manifestation of gravitropic response through the manipulation of amyloplast sedimentation. It is therefore arguably a tropism indistinguishable from gravitropism, apart from the stimulus by which it is elicited.

## Electrotropism

Electric fields (EFs) are able to elicit bending responses in roots, which is called electrotropism (or galvanotropism). Most studies have been conducted with maize, which responds to EFs above a strength of 0.5 V/cm (Stenz and Weisenseel, 1993; Wawrecki and Zagórska-Marek, 2007). In response to an EF, a bidirectional curvature is formed simultaneously in the TZ and the EZ (**Figure 1**) (Wolverton et al., 2000). Both curvatures take place on the side of the root closest to the anode. Still, because the TZ experiences stimulated growth, while in the EZ growth is inhibited, the bending occurs in opposite directions. The field strength threshold of the EZ response is around 10-fold higher than that of the TZ, while the curvature is up to four times weaker. This results in a stairs-like bidirectional curvature, with a clearly dominant response of the TZ (Stenz and Weisenseel, 1991; Stenz and Weisenseel, 1993; Wolverton et al., 2000). Root electrotropism in the few tested species is directed away from the anode and toward the cathode (Stenz and Weisenseel, 1991; Wolverton et al., 2000). As this aligned with the conventional current flow and therefore the direction of the electric field, the response can be called cathodal or negative electrotropism (i.e., growth away from the direction of the electrical stimulus).

Many practical problems have been encountered in the study of electrotropism and the underlying mechanisms are still unknown (Wawrecki and Zagórska-Marek, 2007). In early studies on maize, high strength EFs up to 63 V/cm were used (Ishikawa and Evans, 1990b). Serious damage on the anodal side of the root then causes the root to bend toward the anode in a seemingly positive electrotropism (Stenz and Weisenseel, 1993). Later research indicated that the threshold for damage-induced growth alteration lies at 2–3 V/cm EF strength for maize, depending on the medium (Stenz and Weisenseel, 1991; Stenz and Weisenseel, 1993). This is not uniform across species, as cress (*L. sativum*) can withstand stronger EFs and Black gram (*Vigna mungo*) EFs up to 25 V/cm (Stenz and Weisenseel, 1991; Wolverton et al., 2000).

Similar to experiments with chemotropism, some studies indicated that electrotropism could be a combination of physical effects rather than a true adaptive growth response. For instance, damage to the root apical meristem (RAM) architecture causes formation of a new root cap just above the response threshold of 0.5 V/cm in maize (Wawrecki and Zagórska-Marek, 2007). At 1.0 V/cm root columella initials show decreased division rates, while accumulating starch granules (Wawrecki and Zagórska-Marek, 2007). This indicates that roots are being damaged, even when negative electrotropism is observed. Wolverton et al. (2000) also noted the similarity between the observed TZ hyperpolarization in electrotropism and during gravitropism (Ishikawa and Evans, 1990b). It is therefore possible that electrotropism is due to a combination of root damage and electrical/magneto stimulation of the gravitropic mechanism.

## Thermotropism

Research into possible thermotropism i.e., redirection of growth in response to a temperature gradient, peaked around the turn of the twentieth century. While it was agreed on that the response varied between species, the evidence was often conflicting (Burwash, 1907; Eckerson, 1914; Hooker, 1914; Fortin and Poff, 1991). Almost all indications of thermotropism were of growth toward the warmer side, i.e., positive thermotropism. No consensus on the phenomenon was reached however, and a later publication from this period regarded thermotropism as merely a turgor-driven movement by differential permeability of root cells in different temperatures (Eckerson, 1914).

In 1990 the issue of thermotropism was considered again, providing new evidence for a true tropistic response to thermal gradients in maize (Fortin and Poff, 1990). The threshold for observable thermotropic curvature lies between a 0.5 and 1.4°C/cm horizontal gradient. Maximal thermotropic curvature was obtained in maize under a 4.2°C/cm gradient, with a 15°C starting temperature. Curvature decreased at higher starting temperatures and was absent around 32°C. Contrary to this pattern of curvature, root elongation rates increased continuously up to 26°C (Fortin and Poff, 1990). If thermotropism is merely caused by differences in turgor driven growth experienced by opposite sides of the root, as suggested by Eckerson (1914), it would have been expected to more closely follow the latter pattern instead (Fortin and Poff, 1990). Another publication by Fortin and Poff (1991) further investigated the phenomenon and found indications of a negative thermotropism, i.e., growth away from higher temperatures. Weaker temperature gradients were observed to decrease the maximum temperature at which curvature occurs, besides eliciting smaller angle changes. Thermotropic and gravitropic curvature cancelled each other out when maize roots were gravitropically stimulated (i.e., positioned horizontally) at 19°C, while being exposed to a vertical thermal gradient of 2.6°C/cm. Lower temperatures caused upward curvature of the root, while at higher temperatures gravitropism appeared dominant. With some thermal gradients, a small negative curvature was found at starting temperatures around 40°C (Fortin and Poff, 1991). However, the mechanisms underlying thermotropism remain unknown.

## Oxytropism

Debate on the existence of a tropism toward or away from oxygen, called oxytropism, has remained on the side-lines of tropism research despite promising results. Research on an aerotropism in response to various gasses peaked in the early 1900’s (as reviewed in: Porterfield and Musgrave, 1998). No consensus had been reached on the phenomenon, as a large and systematic investigation by Bennett (1904) disproving aerotropism was challenged. Reports of atypical root growth during research on plants in space prompted Porterfield and Musgrave to revisit the possibility of oxytropism in an Earth-based root growth chamber with gas control, called a microrhizotron (Porterfield and Musgrave, 1998). To this end, wild type pea (*Pisum sativum*) and agravitropic (*ageotropum*) pea mutants were grown in a microrhizotron capable of establishing a 0.8 mmol/mol/mm O_2_ gradient. Curvature of the roots away from the gravity vector and toward higher oxygen concentrations was found at all starting concentrations (26.3–183.8 mmol/mol/mm O_2_) (Porterfield and Musgrave, 1998). Due to the adverse effect of low oxygen concentrations on root elongation, curvature was attenuated at starting concentrations below 131.3 mmol/mol/mm O_2_. While the wild type pea reached 40° bending toward higher oxygen concentrations, the agravitropic mutant was able to reach a full 90°. Despite these promising findings of positive oxytropism, there was no direct follow-up research. Recently however, both an ecological function as well as indications of the mechanism behind oxytropism have been reported for one notable species. Radicles emerging from seeds of the Amazonian floodplain tree *Pseudobombax munguba* grow upwards after germinating submerged in unaerated water (Ferreira et al., 2017). Amyloplasts were absent in these roots, which may be a mechanism to circumvent gravitropism. Aeration of the water column negates this effect, causing radicles with intact amyloplasts to curl and bend downwards. This bending reaction to hypoxia is likely of substantial adaptive value to the *P. munguba* tree seedlings in their search for oxygen when submerged (Ferreira et al., 2017).

Eysholdt-Derzsó and Sauter (2017) documented that hypoxia increased primary root deviations from the vertical in *Arabidopsis*. Under 2% O_2_ concentrations, this deviation reached 38.7° on average, while under 21% O_2_ it was only 14°. Subsequent experiments with mutants of the group VII ETHYLENE RESPONSE FACTORS (ERFVIIs) involved in flooding and hypoxia responses resulted in even more pronounced curvatures under hypoxia. Specifically, mutants of the ERFVII member RAP2.12 reached 70.4° on average. RAP2.12 is stabilized under hypoxia and thought to inhibit hypoxic root bending. Higher auxin levels and lateral auxin asymmetries were detected in the roots of plants bending under hypoxia, pointing toward a mechanism obeying the Cholodny-Went theory. While a decrease of PIN2 protein abundance was also found, it was symmetrical. The authors hypothesize that this contributes to the elevation of auxin in the root tip and the exaggeration of bending (Eysholdt-Derzsó and Sauter, 2017). Both the asymmetric auxin distribution and the decrease in PIN2 abundance could conceivably be guided by an oxygen gradient as stimulus for oxytropism.

## Phonotropism

In recent years, attention for sound as a signal for plants has been increasing. For extensive discussions of the role of sound vibrations in plants, see Mishra et al. (2016) as well as Jung et al. (2018).

Gagliano (2013) proposed a model for sound production in plants involving active organelle movements amplified by the in-phase vibration of neighboring cells. She proposes that sound perception could potentially be achieved through deformation of the plasma membrane and subsequent opening of mechanosensitive (MS) channels. This model was formulated after the dual findings of directional root growth toward sounds and acoustic emission spikes (around 2 m/s) emanating from the *Z. mays* EZ (Gagliano et al., 2012). The directional growth was most noticeable upon 200 and 300 Hz unilateral stimulation of maize roots, with over 40% of roots growing toward the sound source (Gagliano et al., 2012). This reaction, termed phonotropism, was further investigated and K^+^, Ca^2+^, and superoxide were found to be involved (Rodrigo-Moreno et al., 2017).

*Arabidopsis* seedlings formed shorter lateral roots under unilateral 200 Hz stimulation, likely due to increased K^+^ leakage. Five minutes after the start of sound exposure, Ca^2+^ levels increased in the pericycle. Pharmacological evidence indicates the involvement of both plasma membrane Ca^2+^ channels and internal Ca^2+^ release. Subsequent increases in superoxide production were negated by pharmacological inhibition of the plasma membrane Ca^2+^ channels, suggesting an upstream function of Ca^2+^ (Rodrigo-Moreno et al., 2017). While some potential secondary messengers for phonotropism have been identified, the ecological function, if any, is still uncertain. One hypothesis for the ecological relevance of phonotropism is long distance locating of water in soils. Preliminary results show that roots of pea (*P. sativum*) preferentially grow toward the sound of flowing water, which is not in contact with the soil (Gagliano et al., 2017). Attempts to reinforce these findings by playing recorded sound of flowing water were however confounded by potential interference of magnetic fields generated by the equipment (Gagliano et al., 2017).

## Concluding Remarks

Root tropisms are critical for plants, as in nature roots are continuously—and often simultaneously—subjected to multiple stimuli of varying strengths and directions, to which they need to respond to optimize fitness. Note that the term “tropism” must be demarcated carefully, as demonstrated by the cases of chemotropism, electrotropism, and magnetotropism. Tropisms *sensu stricto* are a directional growth response to a directional stimulus. When damaging chemicals, electric- or magnetic fields are applied however, the growth reorientation is arguably not necessarily a growth response, but merely a direct result of inflicted damage. Further investigation on thermotropism, oxytropism, and phonotropism will have to determine if these growth responses are indeed *bona fide* tropisms and what the underlying (molecular) networks are that control these responses.

Crucial to future empirical investigation into tropisms and their and regulation will be the development of experimental tools that enable the study of a single tropism stimulus in isolation, without confounding effects of other environmental gradients of any kind that may elicit a tropistic response. The occurrence of unconfirmed or poorly characterized tropisms, including those stimulated by temperature and oxygen signals, may have significantly influenced the outcomes of published experiments. Caution should be taken therefore, as often no control for such stimuli was in place in published experiments. In addition, many researchers grow their plants on agar medium, with unidirectional light directly reaching the roots when investigating tropistic reactions (Yokawa et al., 2014). Although obviously practical, direct illumination of the roots is known to affect for instance root morphology, hormone reactions, stress response, and even shoot development (Silva-Navas et al., 2015). Covering the roots, for example by use of the “D-root” system should thus be considered for future experimentation on tropisms in order to mitigate confounding light effects (Silva-Navas et al., 2015).

While all tropisms are per definition the result of asymmetric alteration of growth, the initial sensory event of the stimulus varies notably; e.g., from starch filled amyloplasts to phytochromes. For most tropisms, the sensor(s) or even the general sensory tissue(s) are not known (**Table 1**). Between sensing and bending, diverse signal transduction mechanisms and effectors are in place that are partly shared among tropisms induced by diverse stimuli. As already noted by Firn and Myers (1989), this presents a “deceptive unity” that is difficult to disentangle experimentally.

*Arabidopsis* as model organism has proven valuable in the elucidation of several tropism signaling pathways and sensors. In addition, *Arabidopsis* presents significant and underexplored natural variation among accessions in tropistic competence, at least for hydrotropism and phototropism (Sindelar et al., 2014; Vandenbrink and Kiss, 2016; Miao et al., 2018). Exploration of genetic variation can be used in future experiments to elucidate the signal transduction pathways, through genetic analyses by e.g., quantitative trait locus analysis (QTL) or genome-wide association study (GWAS). Nevertheless, focusing on one species has as obvious disadvantage that the pronounced differences in tropisms that exist between species are easily overlooked. Systematic investigations remain therefore important to appreciate the full breadth of variation among species in sensing mechanism, sensor region, stimulus threshold, signaling mechanism, bending direction, and so on. One aspect of the study of root tropism that deserves more attention is the prevalence of tropisms in the natural environment. Apart from scattered examples from e.g., halotropism and oxytropism, there are very few studies examining tropisms outside of the lab environment (Shelef et al., 2010; Ferreira et al., 2017). While lab-based experiments are useful for investigating the molecular and physiological details of the responses, the question remains whether tropisms other than gravitropism play a substantial role in determining root growth direction in a natural setting. In the field, roots are subjected to several opportunities and constraints simultaneously, which all may contribute to the net tropistic response to a certain extent. This also includes allelopathic compounds and other exudates that may also influence parts of the signaling pathway of tropisms (Lupini et al., 2013; Yokawa et al., 2014). Better understanding of the natural occurrence of tropisms would also benefit the translation of lab observations on model species to agricultural field crops and crop improvement strategies (e.g., breeding) (de Dorlodot et al., 2007).

Future experiments on species-specific tropism regulation will benefit strongly from a microgravity environment where gravitropism, being a dominant tropism in many species, can be effectively eliminated. This will enable more detailed studies on weaker tropisms and could help to provide insight into the ecological function of other tropisms.

However, perhaps even more importantly, research on plant tropisms under microgravity environments is indispensable for future space programs. Biological life support systems will be necessary for far journeys into space and to supply moon or planet colonies, that require independent means of subsistence for the astronauts.

In this endeavor, understanding plant tropisms and their changes in a microgravity environment is critical (Zheng et al., 2015), as tropisms need to be controlled to guide the growth of plant roots (and shoots) in altered gravity. For this, knowledge on the “gravitropism masking thresholds” over other tropism and the interactions among tropisms need to be understood in more detail. Research on gravitropism in altered gravity environments has until now largely focused on perception. However, experiments investigating gravitropic signal transduction and response mechanisms are critical for understanding and manipulating root growth at different levels of gravity. After all, between the ISS or a spaceship (µ*g* range), the Moon (0.17*g*), Mars (0.38*g*), and Earth (1*g*), there are magnitude of order differences in the levels of gravity (Kiss, 2015). Without gravitropism dominating as on Earth, it becomes crucial to determine the relative strengths of the different tropisms, in order properly guide root growth by technological means. For example, by exposing roots in microgravity to blue light, they could be induced to develop away from light toward the growth medium. An alternative, particularly suited for reduced gravity environments, is the use of mutants or genome-edited lines with reduced bending responses to establish a more linear root growth, or with increased sensitivity to e.g., gravity. One approach could be to target *WAVY GROWTH 2 (WAV2)*, as WAV2 inhibits root tip rotation and thereby enhances linear growth (Mochizuki et al., 2005). This causes *wav2* mutants to respond to gravity, light, touch, and hydrotropic stimulation with a larger bending angle than wild type *Arabidopsis* (Takahashi et al., 2002; Mochizuki et al., 2005). In this framework, although the use of clinostats proved to be informative for certain parameters, findings will have to be validated in true microgravity environments, necessitating space-based research (Sievers and Hejnowics, 1992; Hoson et al., 1997; Kraft et al., 2000).

Complicating the investigation of tropisms in a microgravity environment such as the ISS, are the changes in plant growth caused by the absence of gravity, that are not related to gravitropism. These changes have for instance been revealed at the cellular and molecular level in biological systems in which tropisms cannot be defined, such as cultured cells *in vitro* (Zupanska et al., 2017; Kamal et al., 2018). Apart from changes in fundamental processes such as cell cycle regulation, ribosome biogenesis, and epigenetics, levels of cytokinin were also altered in microgravity (Ferl and Paul, 2016; Kamal et al., 2018). Additional spaceflight experiments have indicated the occurrence of many other substantial gene expression changes, with many differentially regulated genes connected to pathogen defense and cell wall reorganization (Johnson et al., 2017; Zupanska et al., 2019). Part of these transcriptome changes could influence tropistic functioning as well, changing the behaviour of plant roots in response to stimuli in a space environment. A part of the “spaceflight transcriptome” is in fact dependent on the early gravitropic signaling component ARG1 (Zupanska et al., 2017). Moreover, the ARG1 paralog ARL2 is upregulated in gravitropism and downregulated in response to touch, indicating a complex molecular cross talk between microgravity adaptation and tropistic responses.

Despite several experiments have been performed on tropism interactions, knowledge about the localization of tropistic effects and the signals involved is far from complete (**Tables 1** and **2**). At the same time, identifying secondary messengers can lead to possible identification of new gradients able to establish tropistic bending. For example, ROS are implicated in gravitropism, hydrotropism, and thigmotropism, while also able to control the balance between cell proliferation and differentiation (Tsukagoshi et al., 2010). Similarly, flavonols, forming a gradient during negative phototropism, are able to influence auxin signaling, ROS content, and the meristem regulating PLETHORA protein gradient (Galinha et al., 2007; Silva-Navas et al., 2016). In this regard, the accumulated wealth of information from gene expression studies holds a potential for the identification of new, or shared, signaling components which could be pursued (Kimbrough et al., 2004; Salinas-Mondragon et al., 2010; Strohm et al., 2014; Toal et al., 2018). Other important prerequisites include information about the response ranges and their relation to stimulus strength combined with knowledge about the relative strength of tropisms when occurring simultaneously. Especially considering the need for compensation of gravitropism in microgravity conditions, better understanding of the interactions among tropisms is necessary.

Literally and figuratively back on Earth, the Cholodny-Went theory of differential auxin distribution still stands firm as the starting point into many investigations of tropisms, as it remains the dominant theory for explaining root tropisms, while nearing its 100-year anniversary. At the same time, however, it becomes increasingly clear that the Cholodny-Went theory is not generally applicable to all root tropism responses to diverse environmental stimuli. Future research therefore will have to refine the theory and further determine commonalities and differences in the molecular and physiological processes orchestrating root tropisms, before efficient translation to microgravity and reduced gravity situations can be made.

## Author Contributions

LM, LI, and GA contributed conception and design of the review. LM organized the reference database and wrote the draft of the manuscript. LI, MZ, and GA contributed to manuscript writing and revision. LM and LI designed the figure and tables. All authors read and approved the submitted version and declare no competing interests.

## Conflict of Interest

The authors declare that the research was conducted in the absence of any commercial or financial relationships that could be construed as a potential conflict of interest.
